# MDAN-21: A Bivalent Opioid Ligand Containing mu-Agonist and Delta-Antagonist Pharmacophores and Its Effects in Rhesus Monkeys

**DOI:** 10.1155/2012/327257

**Published:** 2012-04-29

**Authors:** Mario D. Aceto, Louis S. Harris, S. Stevens Negus, Matthew L. Banks, Larry D. Hughes, Eyup Akgün, Philip S. Portoghese

**Affiliations:** ^1^Department of Pharmacology and Toxicology, School of Medicine, Virginia Commonwealth University, Richmond, VA 23298, USA; ^2^Department of Medicinal Chemistry, College of Pharmacy, University of Minnesota, Minneapolis, MN 55455, USA

## Abstract

MDAN-21, 7′-{2-[(7-{2-[({(5*α*, 6*α*)-4,5-Epoxy-3,14-dihydroxy-17-methylmorphin-6-yl}-aminocarbonyl)metoxy]-acetylamino}-heptylaminocarbonyl)-methoxy]-acetylamino}-naltrindole, a bivalent opioid ligand containing a mu-opioid receptor agonist (derived from oxymorphone) linked to the delta-opioid receptor antagonist (related to naltrindole) by a spacer of 21 atoms, was reported to have potent analgesic properties in mice. Tolerance, physical dependence, and conditioned place preference were not evident in that species. The finding that bivalent ligands in this series, with spacers 19 atoms or greater, were devoid of tolerance and dependence led to the proposal that MDAN-21 targets heteromeric mu-delta-opioid receptors. The present study focused on its effects in nonhuman primates (*Macaca mulatta*), a species with a physiology and behavioral repertoire not unlike humans. With regard to opioids, this species usually better predicts clinical outcomes. MDAN-21 substituted for morphine in morphine-dependent monkeys in the remarkably low dose range 0.006–0.032 mg/kg, subcutaneously. Although MDAN-21 failed to produce reliable thermal analgesia in the dose range 0.0032–0.032 mg/kg, intramuscularly, it was active in the same dose range and by the same route of administration, in the capsaicin-induced thermal allodynia assay. The results suggest that MDAN-21 may be useful in the treatment of opioid dependence and allodynia. The data provide additional evidence that opioid withdrawal is associated with sensitized pain.

## 1. Introduction

The alkaloid morphine has been used in the treatment of pain, cough, and diarrhea. Unfortunately, unpleasant and/or potentially dangerous side effects such as respiratory depression, nausea, vomiting, and constipation can accompany its use. Psychological and physiological processes such as abuse, tolerance, and physical dependence have been associated with chronic use and limit the utility of morphine and other mu-opioid agonists in the treatment of chronic pain.

**Figure 1:**
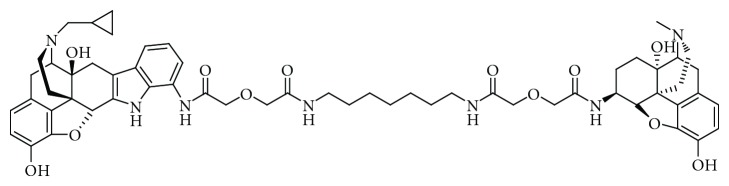
Chemical structure of MDAN-21.

In a continuing search for potent analgesics free of these undesirable side effects, many analogues of morphine and numerous semisynthetic and synthetic derivatives have been introduced. The evolution of this search was summarized in a succinct review of the exciting but vain quest for the Holy Grail of opioid research [1]. Results of an investigation of the effects of leucine and methionine enkephalin on morphine-induced analgesia suggested an interaction between mu- and delta-opioid receptors [2]. Over a decade later, investigators found that naltrindole, a selective delta-opioid receptor antagonist, blocked the morphine tolerance without diminishing its antinociceptive potency [3]. These reports and the finding that G-protein-coupled opioid receptors existed as heterodimers: mu-kappa [4], mu-delta [5, 6], and kappa-delta [7] led to a hypothesis-driven synthesis [8] of a series of conjugates containing the mu-opioid receptor agonist (derived from oxymorphone) linked with delta-opioid receptor antagonist (related to naltrindole) with spacers ranging from 16 to 21 atoms [9]. Studies in mice indicated that 7′-{2-[(7-{2-[({(5*α*, 6*α*)-4,5-Epoxy-3,14-dihydroxy-17-methylmorphin-6-yl}-aminocarbonyl)-metoxy]-acetylamino}-heptylaminocarbonyl)-methoxy]-acetylamino}-naltrindole or MDAN-21, (see Figure 1), with a 25.4 Å spacer, (21-atoms), was the most potent analgesic of the series. Significantly, naloxone, an opioid receptor antagonist, failed to precipitate withdrawal signs after chronic administration. It was subsequently shown that MDAN-21-treated mice failed to develop conditioned place preference [10]. These properties suggested that MDAN-21 may, to a significant extent, fulfill the long quest for a strong opioid analgesic with greatly reduced side effects. We decided to determine MDAN-21's effects on morphine-induced physical dependence and explore its analgesic profile in nonhuman primates. The rhesus monkey study models were selected because of their excellent relationship to the behavioral expressions of opioid effects in humans and their predictive value regarding pharmacotherapy [11] especially regarding opioids.

## 2. Material and Methods

### 2.1. Subjects

Male and female rhesus monkeys (*Macaca mulatta*) served as subjects. All monkeys had prior exposure to drugs and to the behavioral procedures in which they were tested. The subjects in the substitution of morphine assay were housed in groups, whereas those in the thermal nociception and capsaicin-induced thermal allodynia assays were individually housed. Food and water were freely available. Their diet consisted of Purina Lab Diet Fiber-Plus Monkey Biscuits no. 5049 (PMI Feeds, Inc., St. Louis, MO) supplemented with fresh fruit twice weekly. A 12 h light/12 h dark cycle was in effect (lights on from 7AM–7PM).

Animal maintenance and research were conducted in accordance with the guidelines provided by the NIH Committee on Laboratory Animal Resources. The facility was licensed by the United States Department of Agriculture and accredited by the Association for the Assessment and Accreditation of Laboratory Animal Care. The protocols were approved by the Institutional Animal Care and Use Committee. The health of the monkeys was monitored daily by technical and veterinary staff. Monkeys had visual, auditory and olfactory contact with other monkeys throughout the study. Monkeys also had access to puzzle feeders, mirrors, and chew toys to provide environmental enrichment. Nature videotapes or music were played at least twice a week in all housing rooms.

### 2.2. Substitution for Morphine Assay

Twenty-four monkeys in the weight range 3.5–7.5 kg comprised the group tested. The assay was based on that originally described by Deneau [12]. Modifications follow. Morphine was given subcutaneously (s.c.) daily at 6 AM, 12 noon, and 6 PM. All the animals had received morphine for at least 3 months and were maximally dependent on morphine. At least 4 monkeys/treatment were used and a minimal two-week wash-out period was allowed between testing. The assay was modified as indicated below [13, 14]. It was initiated by the injection (s.c.) of the test drug or control substances (morphine and vehicle) into animals in a group that had not received morphine for 14-15 hr and showed definite signs of withdrawal. Each animal was randomly chosen to receive one of the following treatments: (a) a dose MDAN-21; (b) morphine sulfate control (4.0 mg/kg); (c) vehicle control (1.0 mL/kg). Withdrawal signs were scored, absent, or present, once during each of five consecutive 30-min observation periods. Withdrawal signs included slowing of movement, drowsiness (sitting with eyed closed and lethargic or being indifferent to surroundings), fighting, vocalizing, rigidity of abdominal muscles, vocalization during palpation of abdominal muscles, restlessness (pacing), tremors, coughing, retching, vomiting, wet-dog shakes, and masturbation. The observer was “blind” regarding the assignment of treatments. The cumulative number of withdrawal signs was analyzed using the Kruskal-Wallis Analysis of Variance (ANOVA) and post hoc Mann-Whitney *U* Tests. The Stat View statistical package (Brainpower, Agoura Hills, CA) was utilized for these analyses. In all cases, significance (*P*) was set at 0.05.

### 2.3. Assay of Thermal Nociception

 Three adult rhesus monkeys were studied in this assay as described previously [15]. They were seated in acrylic restraint chairs so that their tails hung down freely. During tail-withdrawal measurements, the bottom 15 cm of each monkey's shaved tail was immersed in a thermal container of warm water. If the subject did not withdraw its tail within 20 s, the tail was removed from the water by the experimenter, and a latency of 20 s was assigned to that measurement. Experiments were conducted no more than once a week. A stopwatch was used to measure and record time intervals. Each test session consisted of multiple cycles. Before MDAN-21 administration, baseline tail-withdrawal latencies from 38 and 50°C water were determined. Testing continued only if tail withdrawal from 38°C water did not occur before the 20 s cutoff, and if tail withdrawal occurred in ≤2 s from 50°C water. All monkeys met this criterion before every test session. The effects of MDAN-21 intramuscularly (i.m.) were evaluated using a time-course procedure, in which a single dose of MDAN-21 (0.0032–0.32 mg/kg) was administered at the start of the session, and tail withdrawal latencies from 50°C water were redetermined 3, 6, 10, 18, 32, 56, and 100 min after the injection.

MDAN-21 was completely ineffective up to the highest doses tested in two monkeys. Accordingly, a follow-up experiment was conducted in these two monkeys to determine whether MDAN-21 might function as an antagonist of the high-efficacy mu-agonist methadone. For these studies, monkeys were pretreated with vehicle or 0.32 mg/kg MDAN-21 15 min before treatment with 0.32 mg/kg methadone. Tail-withdrawal latencies were then determined 10, 20, and 30 min after methadone administration. Drug effects were expressed as Percent Maximum Possible Effect (%MPE) using the following equation: “%MPE = (Test Latency − Baseline Latency)/(20 − Baseline Latency)∗100”, where “Test Latency” was the tail-withdrawal latency from 50°C water obtained during each cycle after drug administration, “Baseline Latency” was the latency from 50°C water during the baseline determinations before drug injection, and “20” was the cutoff latency in s.

### 2.4. Assay of Capsaicin-Induced Thermal Allodynia

Three different male rhesus monkeys 5–12 kg were studied. Monkeys were seated in acrylic restraint chairs as described above. To determine tail-withdrawal latencies, the lower 15 cm of each monkeys shaved tail was immersed into a thermal container of warm water heated to the designated temperature (see below for temperatures). The latency in seconds for the monkey to remove its tail from the water was measured using a hand-held stopwatch. If the subject did not withdraw its tail within 20 s, the tail was removed from the water by the experimenter, and a latency of 20 s was assigned to that measurement. Experimental sessions were conducted once per week. At the beginning of each session, tail withdrawal latencies were determined for each monkey from water heated to 38, 42, 46, and 50°C, and the order of temperature presentations was randomized across sessions. By this procedure, baseline temperature-effect curves were determined in each monkey at the beginning of each session, and the highest temperature to produce a tail withdrawal latency >15 s was identified. Water heated to this “threshold” temperature then served as the thermal stimulus for subsequent studies of allodynia during that session. The threshold stimulus intensity was 42°C for two monkeys and 46°C for the third monkey throughout the study.

Allodynia was elicited by topical application of capsaicin as described previously [15, 16]. Following baseline tail withdrawal latency determinations, a topical patch treated with capsaicin solution or vehicle was prepared as described below (see Drugs), and the patch was applied to a region approximately 7 cm from the bottom of the tail for 5 min. After 5 min, the patch was removed. Tail withdrawal latencies were then redetermined 15, 30, 45, and 60 min after patch removal using the thermal stimulus identified from the baseline temperature-effect curve in each monkey (i.e., 42°C in two monkeys, 46°C in the third monkey). MDAN-21 (0.0032–0.32 mg/kg, i.m.) was administered 15 min before application of the capsaicin patch. Morphine (1.0 mg/kg, i.m., 15 min pretreatment) was tested for comparison.

Raw tail withdrawal latencies obtained 15, 30, 45, and 60 min after removal of the capsaicin patch were converted to Percent Maximum Possible Effect (%MPE) using the equation %MPE = [(Test Latency − Capsaicin Alone Latency)/(20 − Capsaicin Alone Latency)∗100)], where “test latency” was the tail withdrawal latency obtained at each time point after drug pretreatment + capsaicin patch treatment, and “Capsaicin Alone Latency” was the latency obtained at the corresponding time point after treatment with the capsaicin patch alone.

### 2.5. Drugs

MDAN-21 was synthesized by Dr. Eyup Akgun (Medicinal Chemistry Laboratory, Dr. Philip Portoghese, Director). Morphine sulfate was purchased from Mallinckrodt, Inc., Hazelwood, MO. Methadone HCl was provided by the NIDA Drug Supply Program. All drugs were dissolved in sterile water for injection (Hospira, Inc., Forest Hills, IL). Capsaicin (Sigma Chemical Co., St. Louis, MO) was dissolved in vehicle composed of 70% alcohol and 30% sterile water and was delivered transdermally (topical patch) as described previously [15, 16]. The concentration of capsaicin in the solution was individually determined for each monkey as the lowest concentration to produce sustained decreases in tail-withdrawal latencies from the threshold temperature to ≤5 s throughout the 1 hr testing period (0.61 mg/mL (2 mM)) for all three monkeys in the study. Within 30 s of preparing the capsaicin patch, it was secured onto the monkey's tail with elastic tape and left on for 5 min.

## 3. Results

### 3.1. Substitution for Morphine in Morphine-Dependent Rhesus Monkeys Assay

**Figure 2:**
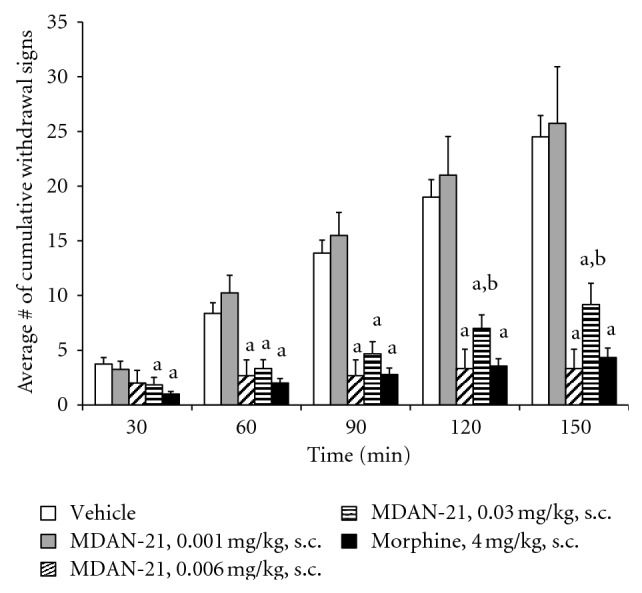
Effects of MDAN-21, morphine, and vehicle in morphine-dependent monkeys in spontaneous withdrawal. ^a^Significantly less (*P* < 0.05) than vehicle and 0.001 mg/kg of MDAN-21. ^b^Significantly less (*P* < 0.05) than MDAN-21. Each point shows mean ± S.E.M. from at least 4 monkeys.

Results of this assay are illustrated in Figure 2. It is evident that MDAN-21 had a short onset and long duration of action, that is, it effectively suppressed withdrawal as soon as 30 min and was still effective at 150 min. MDAN-21 and morphine suppressed the full spectrum of withdrawal signs exhibited by the vehicle controls. The effective dose range was 0.006–0.03 mg/kg. The Kruskal-Wallis ANOVA *P* values are as follows: 30 min-0.01; 60 min-0.0002; 90 min-0.0002; 120 min-0.0001; 150 min-0.0001. Post hoc Mann-Whitney comparisons with probability values of 0.05 or less are indicated in figure legends and with appropriate superscript letters in Figure 2.

### 3.2. Assay of Thermal Nociception

**Figure 3:**
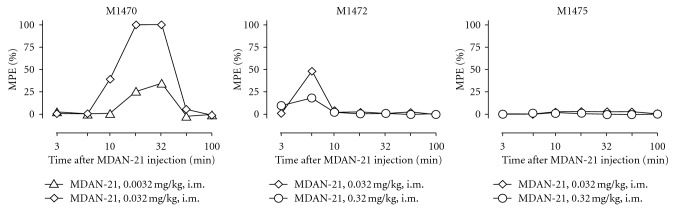
Effects of MDAN-21 in individual monkeys tested in a warm-water tail-withdrawal assay of thermal nociception. Abscissae: minutes after administration of MDAN-21 (log scale). Ordinates: percent maximum possible effect (%MPE). The identification number of the monkey is shown in the upper left of each panel.

The mean ± S.E.M baseline tail withdrawal latency from 50°C was 0.75 ± 0.18 s.  Figure 3 shows the time course of antinociceptive effects produced by doses of 0.0032–0.32 mg/kg MDAN-21 in three individual monkeys. In monkey M1470 (one of the two males), MDAN-21 doses of 0.0032 and 0.032 mg/kg produced a dose-dependent increase in tail withdrawal latencies that peaked after 18 and 32 min and then dissipated after 56 min. However, in the other two monkeys, MDAN-21 doses of 0.032 and 0.32 mg/kg were largely ineffective, producing only small increases in tail-withdrawal latencies after 6 min in monkey M1472. Higher doses were also tested in M1472 (2.6 mg/kg) and M1475 (1.0 mg/kg) and these doses were also ineffective (maximum effect of 15% MPE at any time in either monkey; data not shown).

**Figure 4:**
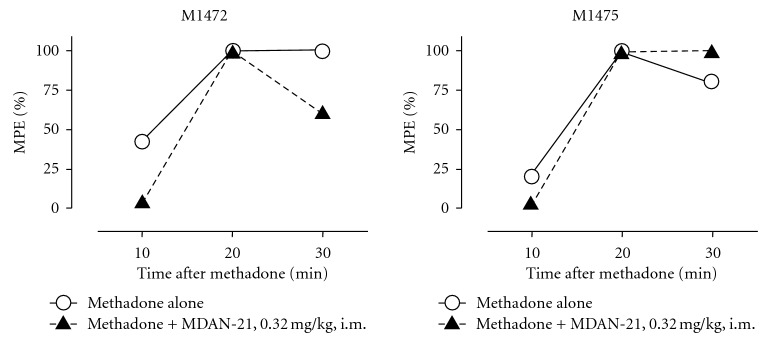
Antinociceptive effects of methadone administered alone or after pretreatment with MDAN-21 in monkey M1472 (left panel) and M1475 (right panel). Abscissae: minutes after methadone administration. Ordinates: percent maximum possible effect. Each point shows the results from a single determination in each monkey.

Figure 4 shows the effects of pretreatment with vehicle or MDAN-21 on the antinociceptive effects of 0.32 mg/kg methadone in monkeys M1472 and M1475. After vehicle pretreatment, methadone produced a time-dependent antinociception that peaked after 20–30 min. Pretreatment with 0.32 mg/kg MDAN-21 had no effect on methadone antinociception in either monkey.

### 3.3. Capsaicin-Induced Thermal Allodynia Assay

**Figure 5:**
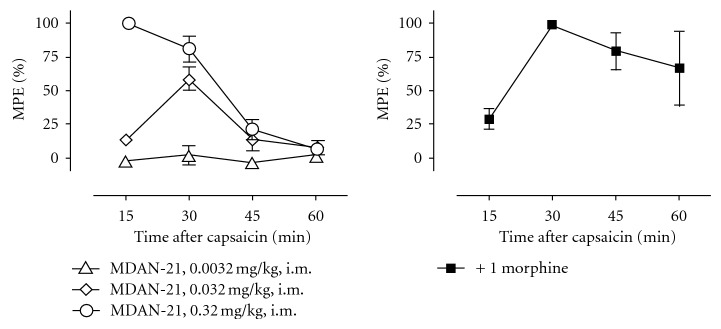
Effects of MDAN-21 (left panel) and morphine (right panel) in an assay of capsaicin-induced thermal allodynia. Abscissae: minutes after removal of the capsaicin patch. Ordinates: percent maximum possible effect. Each point shows mean ± S.E.M. from three monkeys.

The mean ± S.E.M baseline tail withdrawal latency at the threshold temperature was 19.59 ± 0.41 s, and treatment with capsaicin alone reduced tail withdrawal latencies at the threshold temperature to mean ± S.E.M. values of 1.71 ± 0.42, 1. 66 ± 0.23, 1.65 ± 0.41, and 1.49 ± 0.10 s at times 15, 30, 45, and 60 min after capsaicin patch removal, respectively.  Figure 5 shows that pretreatment with MDAN-21 (0.0032–0.32 mg/kg) produced a dose- and time-dependent prevention of capsaicin-induced allodynia. The highest dose of 0.32 mg/kg MDAN-21 produced peak antiallodynic effects after 15 min, and these effects dissipated after 45 to 60 min. For comparison, the antiallodynic effect of 1.0 mg/kg morphine peaked after 30 min and remained at levels greater than 50% MPE in all three monkeys at 45 min and in two of three monkeys at 60 min.

## 4. Discussion

MDAN-21 was very effective in suppressing withdrawal signs in morphine-dependent monkeys. Its action was prompt. It had a remarkably long duration of action compared to that in the capsaicin-thermal assay in nondependent monkeys. Perhaps, MDAN-21 is much more effective in situations involving physical dependence. Increased pain sensitivity in chronic pain patients and opioid abusers has been reported [17–19]. MDAN-21 failed to produce a reliable antinociceptive effect in the assay of thermal antinociception in nondependent monkeys. In one monkey, a dose of 0.032, MDAN-21 was fully effective, but neither this dose nor a 10-fold higher dose of 0.32 mg/kg was active in the other two monkeys. Conversely, MDAN-21 was as effective as morphine in all monkeys tested in the assay of capsaicin-induced thermal allodynia.

This profile of results is consistent with the possibility that MDAN-21 does not readily cross the blood-brain barrier in rhesus monkeys [15]. On the other hand, suppression of withdrawal signs in morphine-dependent monkeys by MDAN-21 suggests that it crosses the blood-brain barrier. It is known that quaternary compounds, do not readily cross the blood-brain barrier. Indeed, quaternary morphine compounds were reported virtually inactive in morphine-dependent rhesus monkeys and practically devoid of antinociceptive activity in mice [20]. The variability in effects produced by MDAN-21 in the assay of thermal nociception did not appear to result from low efficacy of MDAN-21 at mu-opioid receptors. If MDAN-21 had failed to produce antinociception in 2 of 3 monkeys due to low efficacy, then it would be expected to antagonize the effects of a higher efficacy mu-agonist in these two monkeys. However, MDAN-21 failed to antagonize the antinociceptive effects of methadone in either monkey. Nevertheless, it is possible that higher doses might have achieved a robust antinociceptive effect. Finally, species differences in mu-delta interactions on neural substrates that mediate thermal antinociception may be involved [21–23].

Nevertheless, MDAN-21was potently active in the capsaicin-induced thermal allodynia assay in the dose range of 0.032–0.32 mg/kg. It also attenuated withdrawal signs in morphine-dependent monkeys in spontaneous withdrawal for a longer time period. The results are in accord with the findings of other investigators that pain sensitivity is increased in opioid-treated animals, chronic pain patients and opioid abusers (see references above). The results also suggest that MDAN-21 may be useful in the pharmacotherapy of chronic pain and chronic opioid use.
